# Plastic waste as a novel substrate for industrial biotechnology

**DOI:** 10.1111/1751-7915.12312

**Published:** 2015-08-25

**Authors:** Nick Wierckx, M Auxiliadora Prieto, Pablo Pomposiello, Victor de Lorenzo, Kevin O'Connor, Lars M Blank

**Affiliations:** 1 Institute of Applied Microbiology – iAMB, Aachen Biology and Biotechnology – ABBt, RWTH Aachen UniversityWorringerweg 1, Aachen, 52074, Germany; 2 Biological Research Center, The Spanish National Research Council (CIB-CSIC)C/ Ramiro de Maeztu, 9, Madrid, 28040, Spain; 3 BacmineCalle de Santiago Grisolía, Tres Cantos, 28760, Spain; 4 National Center of Biotechnology, The Spanish National Research Council (CNB-CSIC)C/Darwin, 3, Campus of Cantoblanco, Madrid, 28049, Spain; 5 School of Biomolecular and Biomedical Science and Earth Institute, University College DublinBelfield, Dublin 4, Ireland; 6 Bioplastech, NovaUCD, University College DublinBelfield Innovation Park, Belfield, Dublin 4, Ireland

Two hundred and seventy five million tons of plastic waste were produced in 2010 alone (Jambeck *et al*., 2015), with Europe accounting for about 55 million tons per year. The environmental impact of these, primarily fossil-based, plastics has been broadly discussed. While the vast majority of these polymers are not biodegradable, their strength and light weight provide comparative advantages. Poly(ethylene terephthalate) (PET), for instance, has contributed significantly to reducing energy expenditure during transport, especially in the beverage industry. Due to its thermoplastic nature PET is also easy to recycle. However, recycled PET products struggle to compete with virgin PET on price and quality, leading to an overall European recycling rate of less than 30% (PlasticsEurope, 2015). Polyurethanes (PU) are used extensively in a wide range of applications including construction, transportation, furniture and medicine. Since many PU types have a thermoset nature with covalent cross links, one of the main concerns for this plastic is the notable lack of end-of-life recycling (< 5%). Finally, polyethylene (the most used plastic, ca. 140 million tons per year), is considered to be practically inert and its recycling (other than its downcycling into lumber) is economically unfavourable (Sivan, 2011), thereby creating a phenomenal environmental impact, especially in marine environments (Cozar *et al*., 2014; 2015).

While a few countries manage major fractions of plastic waste through incineration in controlled industrial facilities, release of recalcitrant post-consumer plastic into nature remains a major problem globally. Significant amounts of plastic waste contribute to the large-scale pollution of the oceans (Katsnelson, 2015), with terms such as ‘the Great Pacific Garbage Patch’ and ‘the Trash Vortex’ mobilizing public opinion. The widespread distribution of microplastics in the food chain, with as yet unknown effects on biodiversity and human health is also appearing more in the scientific and general literature (Allsopp *et al*., 2006; Setala *et al*., 2014; Avio *et al*., 2015).

The momentum for change away from current practices and towards a sustainable model of exploitation of waste and renewable resources is growing. In order to counteract the pollutant/recycling problems, the revised European Union (EU) Waste Framework Directive has set a minimum plastic recycling target of 50% for household waste and 70% for building and construction waste, which must be reached by all EU member states by 2020. However, without a clear technology roadmap – not to mention an attractive market strategy, the increase in recycling rates will in our opinion not be achievable. Given this background, we propose to use plastic wastes as substrates for the synthesis of added value products, which will empower the recycling industry to a qualitatively new dimension.

How can microbial biotechnology contribute now to this enormous challenge? The advances in our understanding of microbial functions from enzyme, to pathways, and entire metabolic networks now allow the engineering of complex metabolic functions in microbes (Blank and Ebert, 2013). With the ever-advancing tools from synthetic biology e.g. for genome editing (Nikel *et al*., 2014), we can overcome the major challenges for a biotechnological plastic waste-based value chain. Some of the challenges and recent developments are highlighted below.

## Plastic depolymerization

Thermochemical depolymerization of plastics is a rapidly developing field, but often suffers from a lack of added value since the focus lies mostly on liquid and gaseous fuel [synthesis gas (syngas)] production or the re-polymerization to the original polymer. The thermochemical depolymerization of plastic wastes to monomers or syngas, followed by microbial fermentation to generate, for example, higher value biodegradable polymers has emerged as an exciting approach for plastic upcycling (Ward *et al*., 2006; Goff *et al*., 2007; Kenny *et al*., 2008; Guzik *et al*., 2014; Drzyzga *et al*., 2015). However improvements in the growth and levels of biodegradable polymer accumulation by bacteria from these substrates are required. Synthetic biology has the potential to address these plastic waste challenges. Several companies are developing technologies based on syngas fermentation from complex wastes, but most of them are still at the pre-commercial stage since various technical and economic challenges must be overcome before commercial-scale plants can be established (e.g. mass transfer limitation).

Biology may also be able to offer depolymerization solutions. While PET has previously been considered recalcitrant to biological degradation, recently it has been shown that it can be depolymerized by hydrolytic enzymes, such as cutinases and carboxyl esterases, produced by bacteria and fungi (Wei *et al*., 2014). In addition, several bacterial species can use PU as a sole carbon source using enzymes (PUase) mainly classified as esterases, lipases and proteases (Howard, 2002). Hydrolytic enzymes, including members of the mentioned classes, are already produced in bulk quantities and low cost for a variety of applications such as stain-removal agents in detergents and biomass depolymerization in second-generation biorefineries. Hence, the design of novel enzymatic degradation modules employing plastic hydrolases with high rates of plastic depolymerization is possible. While microbes can degrade plastics such as polyethylene, the rates of degradation are slow (Yang *et al*., 2014). One can envisage the emergence of hydrolytic enzymes from nature and advanced genetic engineering with higher rates of depolymerization. In this context, we shall see both natural and tailored plastic-degrading biological agents emerge in the not-so-distant future, which will be able to operate under industrial conditions, where such capabilities can be optimally exploited.

## Novel feedstocks

Besides hydrolytic activities, pathways for efficient use of the plastic monomers arising from depolymerization are required when using plastic waste as a feedstock. The conundrum is similar to issues encountered with lignocellulosic feedstocks, where the engineering of efficient xylose metabolizing strains was a breakthrough technology (for a recent review see: Kim *et al*., 2013). One organism that stands out when it comes to degrading plastic and plastic derived monomers is *Pseudomonas*. This is not surprising in view of the extraordinary metabolic and stress-resistance capabilities growingly found in this type of bacteria (Nikel *et al*., 2014). In fact, this genus includes some of the most efficient PU degraders known, enabling growth rates on polymeric substrates that rival the rates on many conventional substrates (Howard and Blake, 1998). Although *Pseudomonas* cannot degrade PET, its metabolic versatility enables it to utilize terephthalic acid as a carbon source in a biotechnological process for converting PET to polyhydroxyalkanoate (PHA) (Kenny *et al*., 2008). Furthermore, utilization of ethylene glycol, a product of PET depolymerization, was reported for *Pseudomonas* species (Muckschel *et al*., 2012). The exploitation of such catabolic routes for the synthesis of biodegradable polymers has been proposed (O'Connor *et al*., 2013).

## Products from waste plastic

*Pseudomonas* is a promising host for biotechnology with a wide variety of possible products including biodegradable polymers (Ward *et al*., 2006; Goff *et al*., 2007; Kenny *et al*., 2008; Guzik *et al*., 2014; Tiso *et al*., 2015). Specific opportunities may be found in the increasing bioplastic and biosurfactant markets. *Pseudomonas putida* is an excellent choice for biopolymer and biosurfactant production, with reported natural PHA (bioplastic) production of up to 75% of the cell dry weight (Sun *et al*., 2007). In this rapidly developing field, new to nature PHAs can now also be synthesized by engineered microbes (Tortajada *et al*., 2013; Meng *et al*., 2014). These novel polyesters can be co-polymerized *in vivo* or blended during manufacturing, thereby increasing the possible applications. These exciting strategies pave the way to generate high-added value PHAs with applications beyond biodegradable packaging in other fields like nano/biomedicine (Dinjaski *et al*., 2014; Dinjaski and Prieto, 2015). Similarly, biosurfactants such as rhamnolipids can be produced by *Pseudomonas* (Muller *et al*., 2011; Amani *et al*., 2013). Like PHA, the lipid moiety can originate either from lipid *de novo* synthesis or from beta-oxidation or from a combination of both pathways. The reported chain length of the lipid chains vary between 8 and 20 carbon atoms (Abdel-Mawgood *et al*., 2010). In combination with the defined synthesis of mono- and di-rhamnolipids, with one or two rhamnose residues, respectively (Ochsner *et al*., 1995), the synthesis of designer rhamnolipids is possible allowing the production of molecules tailored for a particular application. When choosing a chassis for recombinant rhamnolipid synthesis, two aspects have to be considered. (i) Rhamnolipids are reported to have an antimicrobial activity (Haba *et al*., 2014) and hence the host has to be tolerant against high rhamnolipid concentrations. The Gram positive bacteria *Bacillus subtilis* and *Corynebacterium glutamicum* can be excluded based on this criterion, while *P. putida* grows at a high rate at concentrations above 90 g l^−1^. (ii) Some species of Pseudomonads are (facultative) pathogens, which restrict their application at industrial scale. *Pseudomonas putida* KT2440, having GRAS status, is an attractive host for recombinant rhamnolipid and PHA production (Ochsner *et al*., 1995; Wittgens *et al*., 2011; Tortajada *et al*., 2013).

## Conclusion

Previously thought of recalcitrant waste plastics are now amenable to microbial biotechnology for the production of high-value products relevant to the circular economy. We believe that plastic waste can, and should be established as a novel second-generation carbon source for biotechnology. The analogy to the mega-developments in lignocellulosic biotech is clear: like biomass, plastic waste is simply a carbon-rich polymer. In fact, given the relatively simple and defined composition of plastics compared with biomass, as well as their extreme abundance, it is surprising that this resource has thus far gone mostly unnoticed as a biotechnology feedstock. Parallel developments to produce bio-based versions of fossil-based plastic monomers are already in place, e.g., ethylene, terephthalic acid, ethylene glycol, which will further contribute to the sustainability of plastics. When successful, plastic-upcycling through biotechnology will increase resource efficiency through the valorization of high-volume waste streams, while also helping to reduce the burden on terrestrial resources that are needed to supply food to the world's human population. Thus, through microbial biotechnology, the hereto proposed plastic waste to plastic value workflow will enable new value chains across sectors including materials, chemicals and environmental technologies within the framework of a sustainable knowledge-based bio-economy, with tangible benefits to the environment and society.

## Conflict of Interest

None declared.

## References

[b1] Abdel-Mawgood AM, Lépine F, Déziel E (2010). Rhamnolipids: diversity of structures, microbial origins and roles. Appl Microbiol Biotechnol.

[b2] Allsopp M, Walters A, Santillo D, Johnston P (2006).

[b3] Amani H, Muller MM, Syldatk C, Hausmann R (2013). Production of microbial rhamnolipid by *Pseudomonas aeruginosa* MM1011 for ex situ enhanced oil recovery. Appl Biochem Biotechnol.

[b4] Avio CG, Gorbi S, Milan M, Benedetti M, Fattorini D, d'Errico G (2015). Pollutants bioavailability and toxicological risk from microplastics to marine mussels. Environ Pollut.

[b5] Blank LM, Ebert BE (2013). From measurement to implementation of metabolic fluxes. Curr Opin Biotechnol.

[b6] Cozar A, Echevarria F, Gonzalez-Gordillo JI, Irigoien X, Ubeda B, Hernandez-Leon S (2014). Plastic debris in the open ocean. Proc Natl Acad Sci USA.

[b7] Cozar A, Sanz-Martin M, Marti E, Gonzalez-Gordillo JI, Ubeda B, Galvez JA (2015). Plastic accumulation in the Mediterranean Sea. PLoS ONE.

[b8] Dinjaski N, Prieto A (2015). Smart polyhydroxyalkanoate nanobeads by protein based functionalization. Nanomedicine.

[b9] Dinjaski N, Fernandez-Gutierrez M, Selvam S, Parra-Ruiz FJ, Lehman SM, Roman JS (2014). PHACOS, a functionalized bacterial polyester with bactericidal activity against methicillin-resistant *Staphylococcus aureus*. Biomaterials.

[b10] Drzyzga O, Revelles O, Durante-Rodríguez G, Díaz E, García JL, Prieto A (2015). New challenges for syngas fermentation: towards production of biopolymers. J Chem Technol Biotechnol.

[b11] Goff M, Ward PG, O'Connor KE (2007). Improvement of the conversion of polystyrene to polyhydroxyalkanoate through the manipulation of the microbial aspect of the process: a nitrogen feeding strategy for bacterial cells in a stirred tank reactor. J Biotechnol.

[b12] Guzik MW, Kenny ST, Duane GF, Casey E, Woods T, Babu RP (2014). Conversion of post consumer polyethylene to the biodegradable polymer polyhydroxyalkanoate. Appl Microbiol Biotechnol.

[b13] Haba E, Bouhdid S, Torrego-Solana N, Margués AM, Espuny MJ, Garcia-Celma MJ (2014). Rhamnolipids as emulsifying agents for essential oil formulations: antimicrobial effect against *Candida albicans* and methicillin-resistant *Staphylococcus aureus*. Int J Pharm.

[b14] Howard GT (2002). Biodegradation of polyurethane: a review. Int Biodeterior Biodegradation.

[b15] Howard GT, Blake RC (1998). Growth of *Pseudomonas fluorescens* on a polyester-polyurethane and the purification and characterization of a polyurethanase-protease enzyme. Int Biodeterior Biodegradation.

[b16] Jambeck JR, Geyer R, Wilcox C, Siegler TR, Perryman M, Andrady A (2015). Plastic waste inputs from land into the ocean. Science.

[b17] Katsnelson A (2015). News feature: microplastics present pollution puzzle. Tiny particles of plastic are awash in the oceans – but how are they affecting marine life?. Proc Natl Acad Sci USA.

[b18] Kenny ST, Runic JN, Kaminsky W, Woods T, Babu RP, Keely CM (2008). Up-cycling of PET (polyethylene terephthalate) to the biodegradable plastic PHA (polyhydroxyalkanoate). Environ Sci Technol.

[b19] Kim SR, Park YC, Jin YS, Seo JH (2013). Strain engineering of *Saccharomyces cerevisiae* for enhanced xylose metabolism. Biotechnol Adv.

[b20] Meng DC, Shen R, Yao H, Chen JC, Wu Q, Chen GQ (2014). Engineering the diversity of polyesters. Curr Opin Biotechnol.

[b21] Muckschel B, Simon O, Klebensberger J, Graf N, Rosche B, Altenbuchner J (2012). Ethylene glycol metabolism by *Pseudomonas putida*. Appl Environ Microbiol.

[b22] Muller MM, Hormann B, Kugel M, Syldatk C, Hausmann R (2011). Evaluation of rhamnolipid production capacity of *Pseudomonas aeruginosa* PAO1 in comparison to the rhamnolipid over-producer strains DSM 7108 and DSM 2874. Appl Microbiol Biotechnol.

[b23] Nikel PI, Martinez-Garcia E, de Lorenzo V (2014). Biotechnological domestication of Pseudomonads using synthetic biology. Nat Rev Microbiol.

[b24] Ochsner UA, Reiser J, Fiechter A, Witholt B (1995). Production of *Pseudomonas aeruginosa* rhamnolipid biosurfactants in heterologous hosts. Appl Environ Microbiol.

[b25] O'Connor K, Kenny S, Nikodinovic J (2013).

[b26] PlasticsEurope (2015). http://www.plasticseurope.org/plastics-industry/market-and-economics.aspx.

[b27] Setala O, Fleming-Lehtinen V, Lehtiniemi M (2014). Ingestion and transfer of microplastics in the planktonic food web. Environ Pollut.

[b28] Sivan A (2011). New perspectives in plastic biodegradation. Curr Opin Biotechnol.

[b29] Sun ZY, Ramsay JA, Guay M, Ramsay BA (2007). Carbon-limited fed-batch production of medium-chain-length polyhydroxyalkanoates from nonanoic acid by *Pseudomonas putida* KT2440. Appl Microbiol Biotechnol.

[b30] Tiso T, Wierckx N, Grunwald P, Blank LM (2015). Non-pathogenic *Pseudomonas* as platform for industrial biocatalysis. Industrial Biocatalysis.

[b31] Tortajada M, da Silva LF, Prieto MA (2013). Second-generation functionalized medium-chain-length polyhydroxyalkanoates: the gateway to high-value bioplastic applications. Int Microbiol.

[b32] Ward PG, Goff M, Donner M, Kaminsky W, O'Connor KE (2006). A two step chemo-biotechnological conversion of polystyrene to a biodegradable thermoplastic. Environ Sci Technol.

[b33] Wei R, Oeser T, Zimmermann W (2014). Synthetic polyester-hydrolyzing enzymes from thermophilic actinomycetes. Adv Appl Microbiol.

[b34] Wittgens A, Tiso T, Arndt TT, Wenk P, Hemmerich J, Müller C (2011). Growth independent rhamnolipid production from glucose using the non-pathogenic *Pseudomonas putida* KT2440. Microb Cell Fact.

[b35] Yang J, Yang Y, Wu WM, Zhao J, Jiang L (2014). Evidence of polyethylene biodegradation by bacterial strains from the guts of plastic-eating waxworms. Environ Sci Technol.

